# Efficacy and safety of Cortex Eucommiae (*Eucommia ulmoides* Oliver) extract in subjects with mild osteoarthritis: Study protocol for a 12-week, multicenter, randomized, double-blind, placebo-controlled trial

**DOI:** 10.1097/MD.0000000000018318

**Published:** 2019-12-16

**Authors:** Hyeon Yeong Ahn, Jae-Heung Cho, Dongwoo Nam, Eun-Jung Kim, In-Hyuk Ha

**Affiliations:** a Jaseng Spine and Joint Research Institute, Jaseng Medical Foundation, Seoul; b Department of Korean Rehabilitation Medicine, College of Korean Medicine, Kyung Hee University, Seoul; c Department of Acupuncture & Moxibustion, College of Korean Medicine, Kyung Hee University, Seoul; d Department of Acupuncture & Moxibustion, College of Korean Medicine, Dongguk University, Gyeongju, Republic of Korea.

**Keywords:** clinical trial, Cortex Eucommiae, *Eucommia ulmoides* Oliver extract, mild osteoarthritis, pain

## Abstract

**Background:**

Osteoarthritis (OA) is a major degenerative disease that affects the elderly. The global prevalence of OA is increasing annually. However, current treatments are unable to halt the progress of OA. At present, pharmacological treatments such as non-steroidal anti-inflammatory drugs (NSAIDs) and cyclooxygenase-2 (COX-2) inhibitors control the pain; however, there may be side effects to these medications. We hypothesized that Cortex Eucommiae (CE; *Eucommia ulmoides* Oliver) extract, which is used as a dietary supplement, may slow down or prevent OA.

**Methods:**

This is a protocol for a 12-week, multicenter, randomized, double-blind, placebo-controlled, parallel-group study to evaluate the efficacy and safety of CE extract in subjects with mild OA. One-hundred subjects with mild OA will be recruited and randomly divided in a 1:1 ratio into 2 groups. One group will receive CE extract for 12 weeks and the other group will receive placebo for 12 weeks. Outcomes will be evaluated by using the visual analog scale (VAS), Korean-Western Ontario and McMaster Universities index (K-WOMAC), Korean-Short Form health survey-36 score (KSF-36), and laboratory test results.

**Discussion:**

This clinical trial is expected to provide evidence of the efficacy and safety of CE extract as a treatment for mild OA.

**Trial registration:**

Clinical Trials.gov NCT03744611, registered on November 12, 2018, at https://clinicaltrials.gov/ct2/show/NCT03744611

## 1 Introduction

Osteoarthritis (OA) is one of the major degenerative diseases that affect the elderly and is characterized by damage to the joints of the body. In OA, the joint cartilage becomes thinner and damaged, which inhibits free movement and causes pain.^[1]^ The global prevalence of OA is increasing annually as a result of the increasing age of the population. Among the elderly, approximately 9.6% of men and 18.0% of women have symptoms of OA.^[2]^ However, the progression of OA cannot be halted with the current treatments. At present, pharmacological treatments, such as non-steroidal anti-inflammatory drugs (NSAIDs) and cyclooxygenase-2 (COX-2) inhibitors, are used to control the pain associated with OA.^[3]^ However, the use of these medications carries a risk of adverse effects.^[4,5]^

*Eucommia ulmoides* Oliver has been used as a traditional herbal medicine in Asia for the treatment of various diseases.^[6]^ The efficacy and safety of *E ulmoides* against various diseases have been reported previously.^[7–11]^ Traditionally, it has been used as a pain remedy in China, and the bark of *E ulmoides* has been used as a treatment for arthritis.^[6,12]^ Recently, the effects and mechanisms of Cortex Eucommiae (CE; *E ulmoides*) extract on OA were studied in vitro, and these studies showed that CE extract inhibited inflammatory mediators.^[13,14]^ In vivo studies have identified the collagen synthesis-promoting and cartilage-protective effects of CE extract and the underlying mechanisms responsible.^[12,15]^ Thus, we will conduct a clinical trial to investigate the efficacy and safety of CE as a treatment for mild OA.

## 2 Methods and analysis

### 2.1 Study design

**Figure 1:**
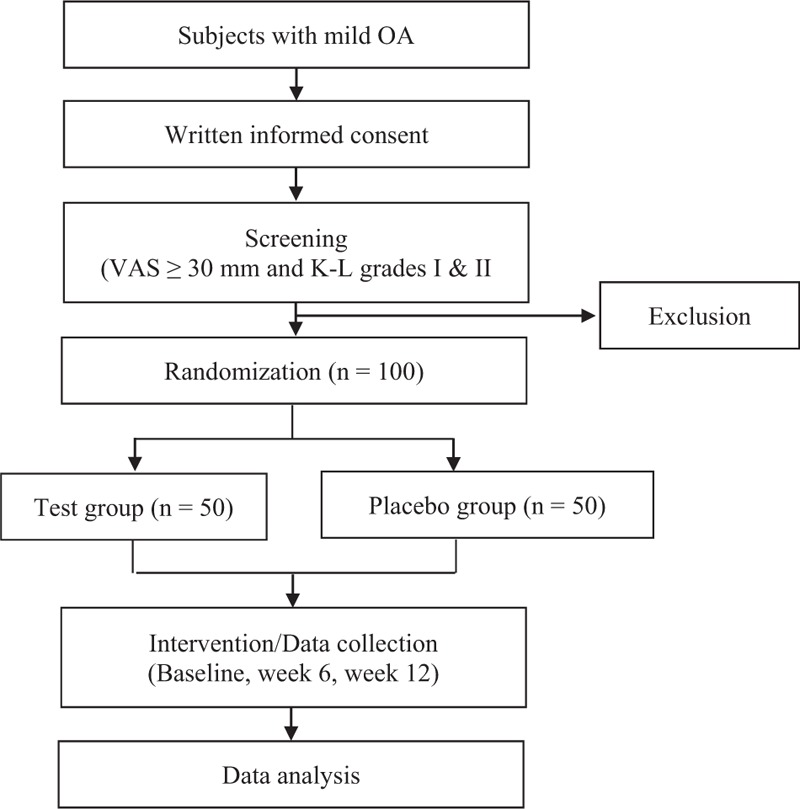
Study flowchart.

This is a 12-week, multicenter, randomized, double-blind, placebo-controlled, parallel-group study. In total, 100 participants will be recruited at Kyung Hee University Korean Medicine Hospital at Gangdong, Kyung Hee University Korean Medicine Hospital, and Dongguk University Bundang Oriental Hospital in Korea. Protocol version is ver.1.4, dated September 06, 2019. Written informed consent will be obtained from each participant before the commencement of any study-related procedure. After written informed consent has been obtained, a screening test will be conducted; subjects meeting all the eligibility criteria will be randomly allocated to either the test group or the control group at a 1:1 ratio. The test group will be instructed to consume the CE extract for 12 weeks and the control group will be instructed to consume the placebo product for 12 weeks. All enrolled subjects will receive guidance on maintaining general diet and activity levels during the study. The participants will visit the hospital four times during the study. Scheduled assessments will be conducted at screening (Week –2 to 0), baseline (Week 0), and at the end of Weeks 6 and 12. The study flow chart is shown in Figure 1.

### 2.2 Recruitment

Participants will be recruited via advertisement to the public. Clinical trial recruitment posters have been posted in hospitals and subways after institutional review board approval. The poster contains brief information about the trial: purpose, method, inclusion criteria, information about the test product, predictable side effects, and information about the participating hospitals and the sponsor.

### 2.3 Participants

#### 2.3.1 Inclusion criteria

Subjects meeting all of the following inclusion criteria may be eligible for enrollment in the study:

- Male or female subjects between 40 and 75 years of age, inclusive
- Subjects with a visual analog scale (VAS) score of more than 30 mm
- Subjects with Kellgren and Lawrence grade I or II determined by X-ray
- Subjects who agree to participate in the clinical trial voluntarily and sign the informed consent form (ICF)

#### 2.3.2 Exclusion criteria

Subjects who meet any of the following exclusion criteria will be excluded from participation in the study.

- Subjects with joint space of less than 2 mm determined by X-ray
- Subjects with Kellgren and Lawrence grade III or higher, with osteophytes, irregularly-shaped auricular surfaces, or subchondral bone cysts visible by X-ray
- Subjects diagnosed with inflammatory arthritis (rheumatoid arthritis, fibromyalgia, systemic lupus erythematosus, septic arthritis, gout), or articulation fracture and an abnormal erythrocyte sedimentation rate (ESR) and C-reactive protein (CRP) level
- Subjects diagnosed with cardiovascular diseases, immune diseases, infectious diseases, and tumors
- Subjects with gastrointestinal diseases
- Subjects with uncontrolled hypertension (blood pressure ≥160/100 mmHg)
- Subjects with uncontrolled diabetes mellitus (fasting glucose level ≥180 mg/dL)
- Subjects with abnormal AST or ALT levels (three times higher than the upper limit of normal)
- Subjects with abnormal creatinine levels (2 times higher than the upper limit of normal)
- Subjects who are pregnant or breastfeeding, or who plan to be pregnant within the next 3 months
- Subjects using OA treatment drugs or dietary supplements in the 2 weeks prior to screening
- Subjects receiving OA treatment therapy in the 2 weeks before screening
- Subjects with psychiatric disorders (schizophrenia or depressive disorder) or who show signs of drug abuse
- Subjects who have participated in another clinical trial in the 3 months prior to screening
- Subjects with a history of hypersensitivity to the investigational product
- Subjects judged as having trial participation-related difficulties by the investigator

#### 2.3.3 Handling of withdrawal and dropout

According to the Declaration of Helsinki, participants may withdraw from the study at any time without providing a reason. Participants may be withdrawn from the study to ensure their safety by the investigator. Investigators may withdraw a participant from the study for any of the following reasons:

- A significant protocol violation
- Occurrence of a serious adverse event (SAE) or adverse event (AE)
- Subject's refusal to participate in the study
- Subject withdraws consent
- Loss to follow-up
- Subject is unable to consume the test or placebo product
- Receipt of any medicine or treatments that could interfere with study participation
- Withdrawal from the study for safety reasons as determined by the investigator
- Pregnancy

### 2.4 Randomization and blinding

The randomization sequence will be generated by Jaseng Spine and Joint Research Institute of Jaseng Medical Foundation, and Microsoft Excel 2010 (Microsoft Inc., USA) will be used to create a blocked randomization list. Randomization codes will be provided in envelopes and kept at each center; randomization will be performed at each center. Eligible participants will be randomly assigned to either the test group or the placebo group in a 1:1 ratio. This study design is double-blind; therefore, both the investigators and the subjects will be blinded during the study. To maintain the blinding, the appearance of the test and placebo product will be identical. The study blind will be broken in situations such as SAEs.

### 2.5 Intervention

One-hundred subjects with mild OA will be recruited and randomly divided into 2 groups; and each subject will receive CE capsules or placebo capsules as dictated by the randomization sequence. Participants will be instructed to consume 1 g/day of *E ulmoides* Oliver extract (2 capsules/day) or placebo (2 capsules/day), by taking 1 capsule in the morning and 1 capsule at night, for 12 weeks. The test and placebo capsules have been manufactured by Nutribiotech Co., Ltd. (Icheon-si, Gyeonggi-do, Korea), a GMP-certified manufacturing facility. The 550 mg test capsule is composed of *E ulmoides* Oliver extract 500 mg, crystalline cellulose 41.75 mg, silica 2.75 mg, and magnesium stearate 5.50 mg. The 550 mg placebo capsule is composed of lactose powder 438.24 mg, caramel color 27.50 mg, purified water 43.59 mg, ethanol 4.40 mg, crystalline cellulose 28.02 mg, silica 2.75 mg, and magnesium stearate 5.50 mg.

### 2.6 Concomitant drugs and therapies

The following drugs, foods, and therapies will be restricted during the intervention period.

- OA medication (eg, Joins tab., etanercept, celecoxib, and hyaluronic acid)
- Aspirin and anti-inflammatory analgesic drugs, corticosteroids, anti-rheumatic drugs
- Penicillamine, diacerein, and pyrazinobutazone
- Cyclophosphamide
- Korean traditional medicinal herbs used to treat OA (eg, Clematidis Radix and Trichosanthis Radix)
- Dietary supplements for joint health (eg, glucosamine, *Perna canaliculus* oil complex, rosehip powder, and *Perilla frutescens*)
- OA therapies (physical therapy, Korean traditional medical treatments such as acupuncture, cupping therapy, and moxibustion)

Information about concomitant drugs and therapies will be recorded in the case report form (CRF).

### 2.7 Outcomes

#### 2.7.1 Primary outcomes

The primary outcomes will be the VAS score, the Korean-Western Ontario and McMaster Universities (K-WOMAC) index, and the Korean-Short Form Health Survey 36 (KSF-36) score.

- VAS will be used to measure changes in joint pain at 6 and 12 weeks after treatment administration and compare it with the baseline score.
- The K-WOMAC index will be used to measure changes in joint pain and function at 6 and 12 weeks after treatment administration and compare it with the baseline index.
- The KSF-36 score will be used to measure changes in physical and mental functions at 6 and 12 weeks after treatment administration and compare it with the baseline score.

#### 2.7.2 Secondary outcomes

- Subject's Global Impression of Change scale score: The change in activity limitations, symptoms, emotions, and overall quality of life, as related to painful conditions, which will be assessed by the subject
- Investigator's Global Impression of Change scale score: The change in activity limitations, symptoms, emotions and overall quality of life, as related to painful conditions, which will be assessed by the investigator
- ESR, to measure changes in blood inflammatory markers at 12 weeks after treatment administration and compare it with baseline ESR
- CRP level, to measure changes in blood inflammatory markers at 12 weeks after treatment administration and compare it with the baseline level

#### 2.7.3 Safety outcomes

The safety outcomes will be AEs, laboratory test results, vital signs (blood pressure and heart rate), weight, and electrocardiography results. An AE is defined as any unexpected occurrence that is not necessarily related to any procedure during the study. The investigator has the responsibility to report all AEs and follow up the AEs until resolved. The relationship between the AEs and the investigational product will be evaluated by the investigator. AE report forms will be completed for all the AEs. The signs and symptoms of the AEs will be described in detail, including the date of onset, duration, severity, action taken, relationship to the investigational product, specific therapy, and outcome. If SAEs occur during the study, the investigator will immediately report the SAEs to the sponsor and the IRB. The following laboratory tests will performed by the clinical laboratory at each center: complete blood count (hemoglobin, hematocrit, and white blood cells [WBC], red blood cells [RBC], platelets, neutrophils, lymphocytes, monocytes, eosinophils, and basophils), blood chemistry tests (blood urea nitrogen [BUN] and levels of aspartate aminotransferase [AST], alanine aminotransferase [ALT], total cholesterol, low-density lipoprotein [LDL]-cholesterol, high-density lipoprotein [HDL]-cholesterol, triglyceride, glucose, total protein, creatinine, uric acid, Na, K, Cl, and Ca), urine tests (specific gravity [SG], pH, and levels of glucose, ketone, bilirubin, urobilinogen, erythrocytes, leukocytes, protein, and nitrite).

### 2.8 Data collection and management

All data collected during the study will be recorded in CRFs. CRFs are kept current to enable the study monitor to review the status of the subjects throughout the course of the study. Data monitoring will be performed by Clinical Research Associate (CRA) of the sponsor. The monitor will review the CRFs and source documents and discuss any missing or spurious data with the investigator. All queries will be resolved in a timely manner by the investigator. The resolution of queries will be recorded in the database. Clean data sets will be provided for statistical analyses and reporting.

The trial schedule is shown in Table 1.

**Table 1:**
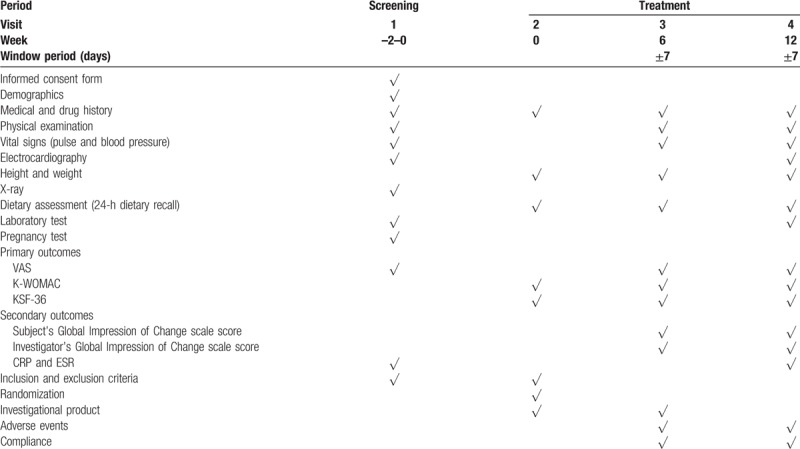
Trial schedule.

### 2.9 Sample size

The sample size was calculated in accordance with the method described by Giordano et al.^[16]^ In their study, the VAS scores for resting pain in the treatment group were significantly decreased from 42.0 ± 24.2 mm to 25.4 ± 19.9 mm (mean ± standard deviation) over 12 weeks. Conversely, the VAS scores for resting pain in the placebo group were increased from 40.9 ± 23.6 mm to 41.0 ± 20.5 mm during the same period. On the basis of these results, we suppose that the change in the treatment group will be –16.6 mm and that in the placebo group will be 0.1 mm. The sample size for this study has been determined using the following parameters:

- Superiority test
- Level of significance, α = 0.05
- Two-sided test
- β = 0.2, power of test = 80%
- Ratio of the number of subjects in the test and placebo groups, λ = 1, n_t_ (subject number of the test group) = λn_c_ (subject number of the placebo group)
- Difference between the groups, Δ = 16.7
- Standard deviation, *σ* = 27.04 (in both groups)

A sample size of 42 subjects per group was calculated, and on the assumption of a dropout rate of 15%, 50 participants will be recruited into each group.

### 2.10 Statistical analysis

Statistical analyses will be computed by using SPSS version 24.0 (SPSS Inc., Chicago, IL). The collected data will be analyzed by using full analysis set analysis, per protocol set analysis, and safety set analysis methods. Missing data will be analyzed by the last observation carried forward method. Baseline characteristics between the test and placebo groups will be evaluated by using the 2-sample *t* test for continuous variables. Categorical variables will be evaluated by using the Chi-square test, Fisher exact test, or McNemar test, and expressed as frequencies and percentages. A paired *t* test will be used to compare the effect within each group both before and after the intervention. ANCOVA and 2 sample *t* tests will be used to compare the net changes between the 2 groups. Skewed variables will be analyzed by using a logarithmic transformation or Wilcoxon rank sum test. Treatment-emergent adverse events (TEAEs) will be coded using the Medical Dictionary for Regulatory Activities (MedDRA) and the percentage of subjects with AEs will be determined and evaluated by using Chi-square test or Fisher exact test. The results will be expressed as the mean ± standard deviation (SD) values and a *P* value of <.05 will be considered to indicate a statistically significant difference.

### 2.11 Ethics and dissemination

The clinical trial was approved by the institutional review board of Kyung Hee University Korean Medicine Hospital at Gangdong (KHNMCOH 2018-07-001), Kyung Hee University Korean Medicine Hospital (KOMCIRB 2018-06-002), and Dongguk University Bundang Oriental Hospital (2018–0008). The study was registered at http://www.clinicaltrials.gov (NCT03744611). Before screening, written informed consent will be obtained from each participant and the investigator will provide each participant with a copy of the signed and dated consent form. All personal information will be maintained confidentiality and only authorized investigator will be able to assess the information. The results will be published in an international peer-reviewed journal.

## 3 Discussion

Most elderly people develop degenerative diseases. OA is a common degenerative disease and its prevalence has been increasing steadily throughout the world.^[17]^ OA is a general type of arthritis, which affects the joints of the knees, hips, lumbar region, shoulders, hands, and feet, and it is a chronic condition in which the joint cartilage becomes thinner and damaged. Consequently, patients with OA experience pain and difficulty in moving freely.^[18,19]^ NSAIDs, COX-2 inhibitors, and other pain killers have been used currently to control the OA-related symptoms and pain. However, most pain medications have side effects. Unfortunately, there are few approaches to slow down OA progression. The best way to treat OA is joint replacement. Therefore, radical treatment methods need to be developed for OA patients.^[20]^

*E ulmoides* Oliver, a member of the Eucommiaceae family, is a traditional herbal medicine in China. It has been used to treat various diseases in Korea, Japan, and China.^[6]^ Various parts of *E ulmoides*, such as the bark, leaf, stem, and flower, have traditionally been used to treat diseases. In addition, *E ulmoides* is nontoxic and has been reported to have few side effects.^[7]^ Previous studies have reported various biological activities of *E ulmoides*, such as antihypertensive, anti-inflammatory, anti-obesity, antitumor, and cardiovascular-protective.^[8–11]^*E ulmoides* has traditionally been used in China to treat back, ankle, and knee pain; in particular, the bark of *E ulmoides* has been used to cure arthritis.^[6,12]^ Recent studies have reported the effectiveness of *E ulmoides* against chronic inflammatory diseases such as OA. Kim et al demonstrated the anti-inflammatory effects of CE, showing inhibition of tumor necrosis factor-α (TNF-α) and interleukin-6 (IL-6) in lipopolysaccharide (LPS)-stimulated mouse macrophages.^[13]^ Koh et al^[14]^ conducted an in vitro study that showed the anti-inflammatory effects of CE in RAW 264.7 cells. CE suppressed inflammatory mediators, such as inducible nitric oxide synthase (iNOS), COX-2, TNF-α, and interleukin-1β (IL-1β), and also modulated the toll-like receptor 4 (TLR-4) pathways, which are expressed in the cartilage of patients with OA.^[14,21]^ The potential of CE as a medicine for chronic inflammatory disease has also been demonstrated. Li et al^[15]^ found the collagen synthesis-promoting effects of CE in an in vivo study. Lu et al^[12]^ demonstrated that elevated serum levels of matrix metalloproteinases (MMPs) are associated with cartilage degradation.^[22]^ The levels of MMP-1, MMP-3, and MMP-13 in the serum decreased in the CE-treated group in the study of Lu et al.^[12]^ Park et al conducted a prospective, randomized, double-blind, multicenter comparative clinical trial to evaluate the efficacy and safety of GCSB-5 compared with those of celecoxib.^[23]^ GCSB-5 is a mixture of 6 herbs and *E ulmoides* Oliver is one of its main components. Patients with knee OA were enrolled and randomly assigned to the GCSB-5 group or the celecoxib group for 12 weeks. The occurrence of AEs or adverse drug reactions was not significantly different between the 2 groups. The total WOMAC and VAS scores improved in both groups. The authors proved the therapeutic effect of GCSB-5 against OA. Therefore, for this clinical trial, we hypothesized that CE may slow down or prevent OA.

This trial has some limitations. First, the subjects may be exclusively Korean. Therefore, the data from this clinical trial cannot be applied to other ethnic groups. Second, owing to the small sample size, the results of this study cannot be generalized. However, few clinical trials have been conducted on the effects of CE on OA. Therefore, despite these limitations, this trial is expected to demonstrate the efficacy and safety of CE for the treatment of patients with mild OA. Furthermore, the results of the study will serve as a foundation for the development of CE as a dietary supplement.

## Author contributions

HYA and IHH planned the study design and wrote the study protocol. JHC, DWN, and EJK reviewed the study protocol. JHC, DWN, and EJK will recruit participants and collect data. HYA wrote the manuscript. All of the authors have read, commented on, and contributed to the submitted manuscript.

In-Hyuk Ha orcid: 0000-0002-5020-6723.
